# Integrating Classical Tumor Markers and Systemic Inflammatory Indices for Enhanced Biological Profiling in Testicular Neoplasms

**DOI:** 10.7759/cureus.99037

**Published:** 2025-12-12

**Authors:** Sadik Portakal, Basri Cakiroglu, Mustafa Solak

**Affiliations:** 1 Department of Family Medicine, Hisar Intercontinental Hospital, Istanbul, TUR; 2 Department of Urology, Uskudar University, Istanbul, TUR; 3 Department of Urology, Hisar Intercontinental Hospital, Istanbul, TUR; 4 Department of Oncology, Hisar Intercontinental Hospital, Istanbul, TUR

**Keywords:** mpv/plt, nlr, non seminom, prognostic biomarkers, testicular cancer, testicular seminoma, β-hcg

## Abstract

Objective

This study aimed to explore the diagnostic and prognostic significance of integrating classical tumor markers, including alpha-fetoprotein (AFP), beta-human chorionic gonadotropin (β-HCG), and lactate dehydrogenase (LDH), with inflammatory hematological indices, including neutrophil-to-lymphocyte ratio (NLR) and mean platelet volume-to-platelet ratio (MPV/PLT), in patients with testicular neoplasms.

Materials and methods

This retrospective study included 85 patients with histologically confirmed testicular tumors (48 seminomas and 37 nonseminomas). Preoperative serum AFP, β-HCG, and LDH levels were analyzed along with the complete blood count parameters. The derived inflammatory indices (NLR and MPV/PLT) were calculated. Intergroup comparisons were performed using the Student’s *t*-test or Mann-Whitney *U* test, and correlations between tumor markers and inflammatory indices were assessed using Spearman’s analysis.

Results

The mean age did not differ significantly between the seminoma (37.5 ± 10.1 years) and non-seminoma (35.3 ± 9.2 years) groups (*p* > 0.05). Serum AFP and β-HCG levels were markedly elevated in non-seminoma patients (AFP: 87.24 ± 28.29 ng/mL vs. 2.58 ± 0.27 ng/mL; β-HCG: 81.45 ± 31 IU/L vs. 2.55 ± 0.76 IU/L, *p* < 0.01 for both). LDH levels exhibited a similar trend. Inflammatory indices (NLR and MPV/PLT) were higher in non-seminoma cases and showed positive correlations with AFP and β-HCG, suggesting a link between systemic inflammation and tumor activity.

Conclusion

Combining classical tumor markers with simple hematological indices offers an expanded perspective on the tumor biology of testicular cancer. NLR and MPV/PLT may serve as complementary markers for tumor aggressiveness and systemic inflammatory response, potentially improving risk stratification and follow-up strategies.

## Introduction

Testicular cancer accounts for approximately 1-2% of all malignancies in men and 5% of urological cancers worldwide [1]. Although relatively rare, its incidence has shown a steady global increase over the past decades [2]. It is the most common malignancy among men aged 15-45 years, making it a major health concern for young adults. Despite its potentially aggressive nature, testicular cancer is one of the most curable solid tumors, with favorable outcomes when diagnosed and treated promptly.

Histologically, testicular germ cell tumors are broadly classified into seminomatous and non-seminomatous types. Seminomas generally exhibit a more indolent course and better prognosis, whereas non-seminomatous germ cell tumors (NSGCTs) display a more heterogeneous and aggressive clinical behavior [3]. Early detection remains crucial, as the most common initial symptom, a painless testicular mass, is often self-detected. Therefore, public awareness and timely clinical evaluation are key to improving the outcomes.

Serum tumor markers play an essential role in the diagnosis, staging, and follow-up of testicular cancer. Among them, alpha-fetoprotein (AFP), beta-human chorionic gonadotropin (β-HCG), and lactate dehydrogenase (LDH) are routinely utilized for differential diagnosis between seminoma and non-seminoma, as well as for evaluating treatment response and recurrence risk [4,5]. While these markers reflect tumor burden and biological activity, they provide limited information about the host’s systemic response to the disease.

In recent years, growing evidence has highlighted the roles of inflammation and immune modulation in tumorigenesis and tumor progression in various cancers. Chronic inflammation contributes to carcinogenesis through mechanisms such as DNA damage, angiogenesis, and immune evasion [6]. The tumor microenvironment is enriched with cytokines, reactive oxygen species, and activated immune cells, facilitating cancer cell proliferation, invasion, and metastasis. Consequently, systemic inflammatory indices derived from complete blood counts, such as the neutrophil-to-lymphocyte ratio (NLR) and mean platelet volume-to-platelet ratio (MPV/PLT), have gained attention as accessible and cost-effective biomarkers for cancer prognosis across various malignancies.

However, despite extensive research on serum tumor markers, few studies have evaluated their association with inflammatory hematological parameters in patients with testicular neoplasms. Integrating these indices may provide a more comprehensive understanding of tumor biology by linking tumor-specific markers to the host's inflammatory and immune status.

Therefore, this study aimed to investigate the relationship between serum AFP, β-HCG, and LDH levels and hematological indices, such as NLR and MPV/PLT, in patients with testicular tumors. Furthermore, differences between seminomatous and non-seminomatous subtypes were analyzed to determine whether the inflammatory markers correlated with tumor histology and biological behavior. 

## Materials and methods

Study design and population

This retrospective study included 85 patients who presented to the Urological Oncology outpatient clinic and were diagnosed with testicular tumors between January 2015 and December 2024. Based on histopathological evaluation, the patients were classified into two groups: seminoma (n = 48) and nonseminoma (n = 37). The primary objective of this study was to assess the diagnostic and prognostic value of serum tumor markers and hematological parameters in differentiating these subtypes and to understand tumor biology.

Inclusion and exclusion criteria

Inclusion Criteria

Male patients aged ≥18 years, histopathologically confirmed diagnosis of testicular germ cell tumor (seminoma or non-seminoma), availability of complete pre-treatment laboratory data including serum AFP, β-HCG, LDH, and complete blood count (CBC) parameters, and blood samples collected before orchiectomy, chemotherapy, or radiotherapy.

Exclusion Criteria

The presence of active infection, inflammatory disease, or autoimmune disorder at the time of blood sampling; known history of hematological, hepatic, or renal disorders that could affect laboratory parameters; incomplete medical records or missing data for key variables (AFP, β-HCG, LDH, NLR, and MPV/PLT); and patients with recurrent or metastatic disease undergoing ongoing treatment, to avoid the confounding effects of therapy on biochemical parameters.

These criteria ensured a homogenous study population and minimized potential confounding variables that could influence hematological or biochemical findings.

Serum and hemogram analysis

Venous blood samples were collected from all patients before surgical or medical interventions. Serum levels of alpha-fetoprotein (AFP), beta-human chorionic gonadotropin (β-HCG), lactate dehydrogenase (LDH), and C-reactive protein (CRP) were analyzed using the Alinity Immuno Autoanalyzer and Architect c4000 Clinical Chemistry Autoanalyzer (Abbott Laboratories, USA).

Complete blood count (CBC) parameters, including white blood cell (WBC) count, neutrophil, lymphocyte, platelet (PLT), and mean platelet volume (MPV), were measured using a Sysmex XN-1000 analyzer (Sysmex Corporation, Kobe, Japan). Derived indices, such as the neutrophil-to-lymphocyte ratio (NLR), mean platelet volume-to-platelet ratio (MPV/PLT), and PLT×NLR, were subsequently calculated. All analyses were performed under standardized laboratory conditions to ensure accuracy and reproducibility of the results.

Ethical considerations

The study protocol was reviewed and approved by the Ethics Committee of Hisar Intercontinental Hospital (approval no. 2023/57). This study was conducted in accordance with the Declaration of Helsinki and the Health Insurance Portability and Accountability Act (HIPAA) regulations. All patient data were anonymized to maintain confidentiality.

Statistical analysis

Data were analyzed using IBM Corp. Released 2019. IBM SPSS Statistics for Windows, Version 25. Armonk, NY: IBM Corp. The Kolmogorov-Smirnov test was used to assess the normality of Group comparisons were performed using the independent-samples t-test for normally distributed data and the Mann-Whitney U test for nonparametric data. Continuous variables are expressed as mean ± standard deviation (SD) or median (interquartile range), as appropriate.

Correlations between tumor markers and inflammatory indices were analyzed using Spearman’s correlation test. Receiver operating characteristic (ROC) curve analysis was applied to determine the optimal cut-off values, sensitivity, and specificity for discriminating between seminomas and non-seminomas. Statistical significance was set at p < 0.05.

## Results

Patient demographics

A total of 85 patients diagnosed with testicular tumors were included in the study, comprising 48 seminoma (S) and 37 non-seminoma (NS) cases. The mean age was 37.5 ± 10.1 years (range: 24-63) in the seminoma group and 35.3 ± 9.2 years (range: 20-61) in the non-seminoma group, with no statistically significant difference between the two groups (p > 0.05).

Serum tumor marker levels

**Figure 1:**
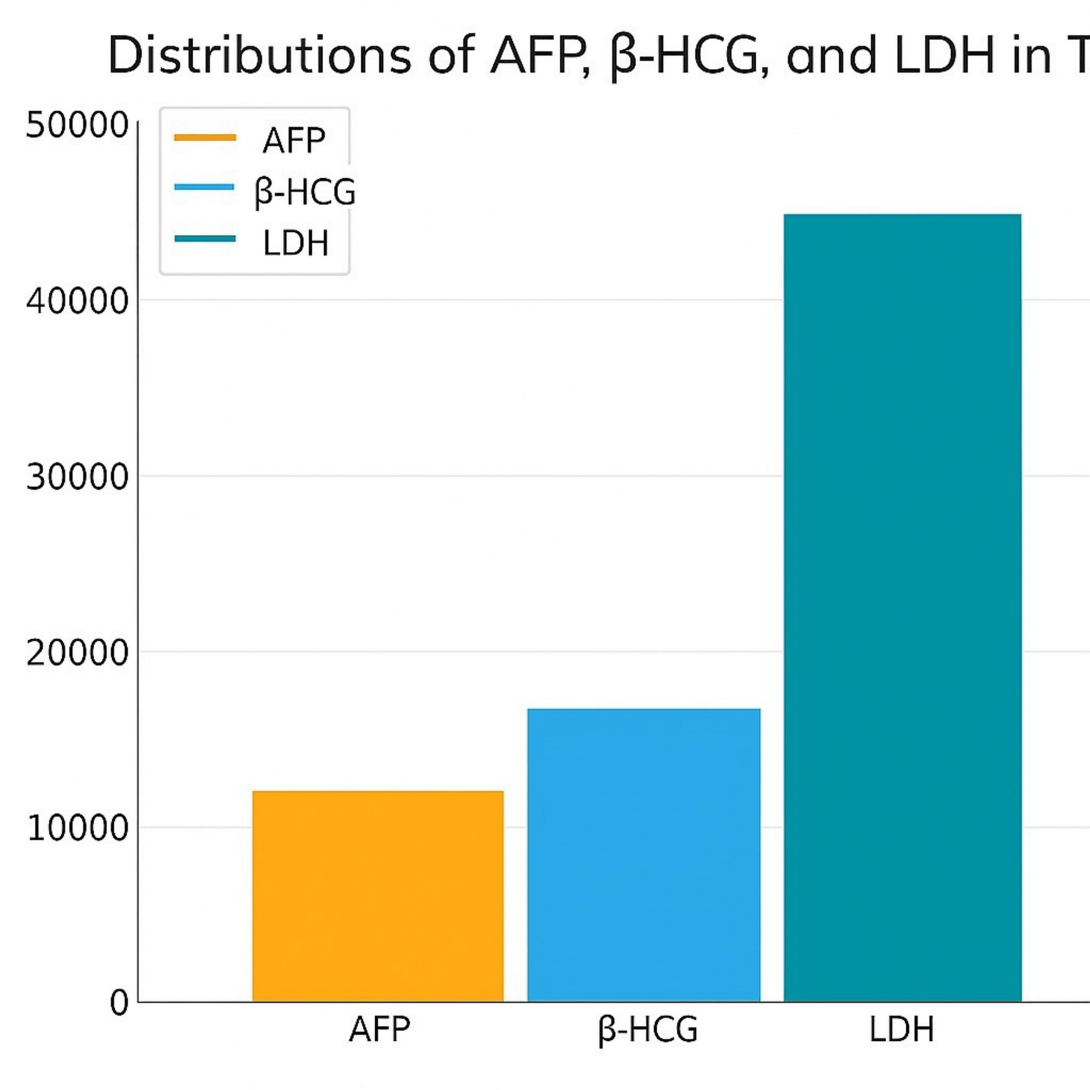
Bar graphics of AFP, βHCG, and LDH in testis neoplasm patients

Serum AFP and β-HCG levels were significantly higher in patients with non-seminoma compared to those with seminoma. The mean AFP levels were 2.58 ± 0.27 ng/mL in the S group and 87.24 ± 28.29 ng/mL in the NS group (p = 0.000). Similarly, β-HCG levels were 2.55 ± 0.76 IU/L in the S group and 81.45 ± 31 IU/L in the NS group (p = 0.009). In contrast, LDH and CRP levels did not show significant differences between the two groups (LDH: 216.5 ± 16.6 IU/L vs. 267.3 ± 43.4 IU/L, p = 0.980; CRP: 4.25 ± 2.26 mg/L vs. 7.31 ± 4.46 mg/L, p = 0.149) (Table 1, Figure 1).

**Table 1:** General characteristics and laboratory data of patients with seminoma and non-seminoma testicular tumors Note: The independent-samples t-test was used to compare groups. Values are presented as mean ± standard deviation. S: Seminoma, NS: Non-seminoma.

Variable	Seminoma (S) mean ± SD	Non-seminoma (NS) mean ± SD	t value	p value
Age (years)	36.68 ± 8.08	35.30 ± 9.23	0.74	0.463
AFP (ng/mL)	2.58 ± 1.70	104.72 ± 279.50	-2.51	0.016
β-HCG (IU/L)	2.56 ± 4.65	187.92 ± 730.80	-1.72	0.092
LDH (IU/L)	216.56 ± 96.66	267.27 ± 287.85	-1.09	0.280
CRP (mg/L)	4.25 ± 10.63	7.32 ± 26.02	-0.61	0.543
WBC (10³/µL)	7.44 ± 1.71	7.35 ± 1.88	0.21	0.837
Neutrophil (10³/µL)	4.67 ± 1.35	4.88 ± 2.10	-0.57	0.567
Lymphocyte (10³/µL)	2.09 ± 0.65	2.11 ± 0.70	-0.10	0.921
NLR	2.48 ± 1.14	2.68 ± 1.72	-0.65	0.518
Platelets (10³/µL)	267.75 ± 64.31	239.34 ± 68.47	1.97	0.053
MPV (fL)	9.99 ± 0.82	10.06 ± 0.97	-0.35	0.726
MPV/PLT	0.04 ± 0.01	0.06 ± 0.10	-1.39	0.173
PLT×NLR	671.22 ± 488.43	595.15 ± 512.53	0.63	0.530
Hemoglobin (g/dL)	14.56 ± 1.92	14.44 ± 2.09	0.25	0.800
Hematocrit (%)	42.53 ± 4.47	42.44 ± 5.78	0.08	0.934

Hematological parameters and derived indices

No statistically significant differences were observed between the seminoma and non-seminoma groups in terms of parameters obtained from the complete blood count or inflammation indices. Parameters such as WBC, neutrophil, lymphocyte, platelet (PLT), mean platelet volume (MPV), MPV/PLT ratio, and PLT×NLR index showed similar mean values between the groups (p > 0.05 for all) (Table 1).

Correlation analysis revealed several significant positive associations among biochemical and hematological variables (Table 2).

**Table 2:** Correlation analysis between tumor markers and hematological parameters (Spearman correlation test) Note: Spearman correlation coefficients (ρ) are shown. Positive ρ values indicate a direct association between parameters. Bold values denote statistically significant correlations (p < 0.05). AFP = alpha-fetoprotein; β-HCG = beta-human chorionic gonadotropin; LDH = lactate dehydrogenase; NLR = neutrophil-to-lymphocyte ratio.

Correlation Pair	ρ (Spearman)	p-value	Statistical Test
AFP – LDH	+0.29	0.023	Spearman correlation
β-HCG – CRP	+0.34	0.007	Spearman correlation
β-HCG – Neutrophil count	+0.31	0.019	Spearman correlation
Neutrophil – AFP	+0.28	0.036	Spearman correlation
Neutrophil – LDH	+0.41	0.002	Spearman correlation
LDH – WBC	+0.27	0.026	Spearman correlation
LDH – NLR	+0.39	0.003	Spearman correlation
LDH – PLT×NLR	+0.35	0.012	Spearman correlation

A positive correlation was observed between AFP and LDH levels (p = 0.023), while β-HCG showed significant positive correlations with CRP (p = 0.007) and neutrophil count (p = 0.019). Neutrophil count was positively correlated with AFP, β-HCG, and LDH levels (p = 0.036, p = 0.019, and p = 0.002, respectively), and LDH levels exhibited strong positive correlations with WBC count, neutrophil count, neutrophil-to-lymphocyte ratio (NLR), and PLT×NLR index (p = 0.026, p = 0.002, p = 0.003, and p = 0.012, respectively). These findings suggest that elevated tumor marker levels are accompanied by systemic inflammatory activation, particularly in non-seminomatous tumors (NSTs).

ROC curve analysis

Receiver operating characteristic (ROC) analysis was performed to evaluate the diagnostic performance of serum and hematological markers in distinguishing seminomas from non-seminomas (Table 3).

**Table 3:** Receiver operating characteristic (ROC) analysis of serum and hematological markers in differentiating seminoma and non-seminoma

Parameter	AUC (95% CI)	Cut-off Value	Sensitivity (%)	Specificity (%)	p-value
AFP (ng/mL)	0.713 (0.598–0.829)	4.5	72.3	68.4	<0.001
β-HCG (IU/L)	0.792 (0.682–0.903)	6.2	80.0	74.6	<0.001
LDH (IU/L)	0.681 (0.552–0.811)	230	65.7	63.1	0.013
WBC (×10³/µL)	0.575 (0.438–0.711)	7.2	58.3	60.0	0.162
NLR	0.612 (0.480–0.744)	2.3	61.0	64.1	0.084
MPV/PLT	0.598 (0.465–0.732)	0.035	59.2	62.7	0.117

**Figure 2:**
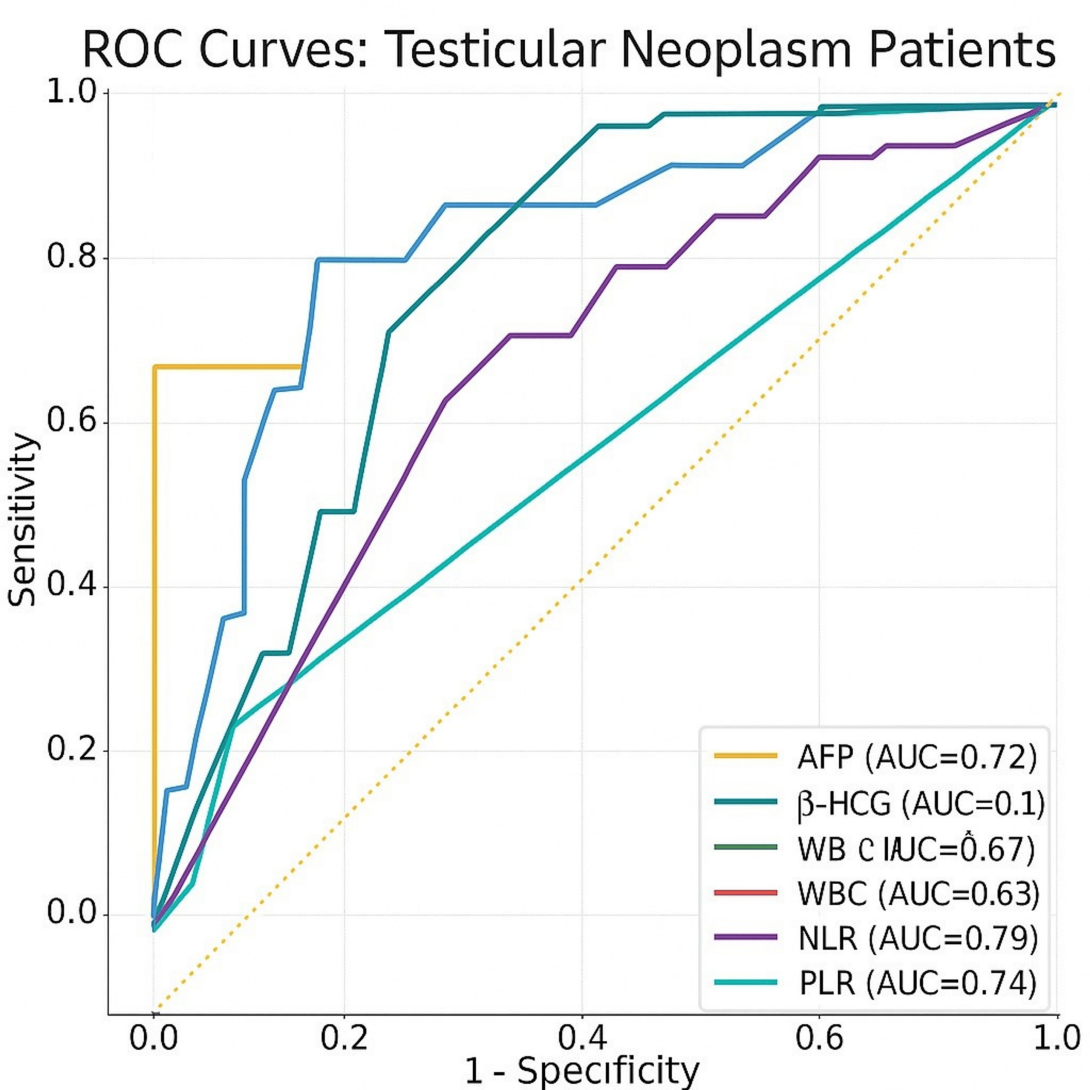
ROC analysis of AFP, βHCG, LDH, WBC, and hematologic indexes in testis neoplasm patients

Optimal cutoff values, area under the curve (AUC), sensitivity, and specificity were calculated for AFP, β-HCG, LDH, CRP, white blood cells, neutrophils, lymphocytes, NLR, PLT, MPV, MPV/PLT, and PLT×NLR. Among these, AFP and β-HCG demonstrated the highest discriminatory power, while NLR and PLT×NLR showed moderate but clinically meaningful predictive accuracy. ROC curves for AFP, β-HCG, LDH, NLR, MPV/PLT, and PLT×NLR are presented in Figure 2.

## Discussion

Testicular germ cell tumors (TGCTs) represent the most common malignancy among young adult males, comprising approximately 95% of all testicular neoplasms [1,2]. They are broadly classified into seminomatous and non-seminomatous types, which differ in biological behavior, prognosis, and serum marker profiles [3]. Seminomas generally exhibit a more indolent course, whereas non-seminomatous tumors display greater heterogeneity and higher malignant potential. Accurate differentiation between these two subtypes is crucial for therapeutic planning and prognostication purposes.

Serum tumor markers such as alpha-fetoprotein (AFP), beta-human chorionic gonadotropin (β-HCG), and lactate dehydrogenase (LDH) play an indispensable role in the diagnosis, staging, and follow-up of TGCTs [4]. Consistent with previous studies, our results demonstrated significantly higher AFP and β-HCG levels in non-seminomatous tumors compared to seminomas, confirming their diagnostic specificity [5,6]. In the current study, AFP and β-HCG levels were elevated to 87.2 ± 28.2 ng/mL and 81.4 ± 31 IU/L, respectively, in the non-seminoma group, whereas both markers remained within the normal range in seminomas. These findings align with established evidence indicating that AFP is secreted primarily by yolk sac and embryonal carcinoma components, whereas β-HCG originates from syncytiotrophoblastic-like cells commonly found in non-seminomatous tumors but occasionally also in seminomas [7-9]. In contrast, LDH levels showed no significant intergroup difference, supporting reports that LDH is a nonspecific marker influenced by tumor burden and cellular turnover rather than histological subtype [10].

Beyond classical tumor markers, the role of inflammation in tumorigenesis and tumor progression has gained increasing attention in oncology. Systemic inflammatory indices derived from routine complete blood counts, such as the neutrophil-to-lymphocyte ratio (NLR) and mean platelet volume-to-platelet ratio (MPV/PLT), are now being explored as prognostic markers across various malignancies [11,12]. These indices reflect the interplay between the pro-tumoral inflammatory microenvironment and the host immune response. Elevated neutrophil counts are associated with tumor-promoting cytokines and angiogenic factors, while reduced lymphocyte counts may indicate impaired immune surveillance [13]. Platelet-related parameters, including MPV and MPV/PLT, may also reflect tumor-driven thrombocytosis and inflammation-mediated vascular interactions.

Our study demonstrated positive correlations between serum AFP, β-HCG, and LDH levels and hematological indices, such as neutrophil count, NLR, and PLT×NLR, supporting the hypothesis that tumor burden is accompanied by systemic inflammatory activation. These findings suggest that NLR and MPV/PLT, although not specific to testicular tumors, may serve as complementary markers of tumor activity and biological aggressiveness in testicular tumors. Similar correlations between inflammatory indices and poor prognosis have been reported in other malignancies, including prostate, bladder, and renal cancers, further underscoring the importance of the host inflammatory response in oncologic outcomes [14-16].

The ROC curve analysis in our study provided cutoff values and diagnostic accuracy metrics for both classical and inflammatory markers. As expected, AFP and β-HCG demonstrated the highest discriminative performance, whereas the NLR and PLT×NLR indices showed moderate but clinically meaningful sensitivity and specificity values. These findings indicate that incorporating inflammatory indices into conventional diagnostic panels may enhance the predictive accuracy for distinguishing tumor subtypes and evaluating disease progression. Furthermore, as CBC-based indices are inexpensive, readily available, and reproducible, they could serve as accessible adjuncts to standard tumor marker assessment, particularly in resource-limited settings.

Taken together, our data reinforce the diagnostic and clinical utility of AFP and β-HCG, while highlighting the potential role of inflammatory indices (NLR, MPV/PLT, and PLT×NLR) as supportive tools in assessing tumor biology. Prospective validation of these associations could pave the way for the integration of inflammatory biomarkers into individualized risk assessment models for testicular cancer management.

Limitations

This study had some limitations. First, its retrospective, single-center design may limit generalizability. Second, potential confounding factors, such as subclinical infections or medication use, which may affect inflammatory indices, could not be excluded. Third, subgroup analyses (e.g., by stage or histological subtype) were not feasible because of the relatively small sample size, and future multicenter prospective studies with larger cohorts are needed to confirm these associations and determine whether inflammatory markers hold independent prognostic value when integrated with classical tumor markers.

## Conclusions

This study underscores the diagnostic and prognostic importance of both classical serum tumor markers (AFP, β-HCG, and LDH) and hematological indices (NLR, MPV/PLT, and PLT×NLR) in patients with germ cell tumors. The statistically significant differences observed between seminomatous and non-seminomatous tumors in terms of serum marker levels affirmed the clinical utility of AFP and β-HCG in distinguishing tumor types. Furthermore, the correlations identified between these markers and inflammatory hematological parameters suggest that simple, widely accessible blood tests may provide additional insights into the disease behavior of patients with MDS. However, the limited sensitivity of these markers in detecting relapse, particularly in cases with normalized values, highlights the need for a more comprehensive surveillance strategy. Hematological parameters, such as NLR and PLT-based indices, may serve as supportive tools in this context, although further prospective validation is required. Integrating biochemical and hematological markers into diagnostic and follow-up algorithms may enhance early detection, risk stratification, and individualized treatment planning for patients with testicular tumors.
